# On Construction of Tibial Plateau Fracture Detection in Different Radiographic Views Using YOLO Models

**DOI:** 10.3390/diagnostics16020182

**Published:** 2026-01-06

**Authors:** Shun-Ping Wang, Han-Ting Shih, Yu-Xiang Liao, Chih-Han Wei, Jung-Chun Liu, Endah Kristiani, Chao-Tung Yang

**Affiliations:** 1 Department of Post-Baccalaureate Medicine, National Chung Hsing University, Taichung City 40227, Taiwan; wsp0120@vghtc.gov.tw; 2 Department of Orthopaedics, Taichung Veterans General Hospital, Taichung City 407219, Taiwan; d13330003@thu.edu.tw; 3 Department of Industrial Engineering and Enterprise Information, Tunghai University, Taichung City 407224, Taiwan; 4 Department of Computer Science, Tunghai University, Taichung City 407224, Taiwan; s10350231@go.thu.edu.tw (Y.-X.L.); s10350239@go.thu.edu.tw (C.-H.W.); jcliu@thu.edu.tw (J.-C.L.); endahkristi@thu.edu.tw (E.K.); 5 Department of Informatics, Krida Wacana Christian University, Jakarta 11470, Indonesia; 6 Research Center for Smart Sustainable Circular Economy, Tunghai University, No. 1727, Sec.4, Taiwan Boulevard, Taichung City 407224, Taiwan; 7 Department of Medical Research, Kuang Tien General Hospital, Taichung City 43304, Taiwan

**Keywords:** artificial intelligence, deep learning, object detection, tibial plateau fracture, X-ray, YOLO

## Abstract

**Background/Objectives:** Tibial plateau fractures are difficult to detect using X-ray imaging due to limited three-dimensional visibility. This study evaluated the performance of four You Only Look Once (YOLO) deep learning models trained on different radiographic views for fracture detection. **Methods:** A total of 1489 knee X-rays were collected from a tertiary referral hospital, comprising 727 fracture images and 762 non-fracture images. YOLOv4, YOLOv5, YOLOv8, and YOLOv9 were each trained using anteroposterior (AP), lateral, and combined views. **Results:** YOLO models trained on AP views consistently outperformed those trained on other views. YOLOv9 trained on AP images achieved the highest accuracy, specificity, precision, F1-score, and area under the curve (AUC) of 0.99, with both sensitivity and negative predictive value (NPV) at 1.00. YOLOv8 trained on AP views reached 0.97 across all metrics with an AUC of 0.98. YOLOv5 trained on AP images achieved an accuracy and F1-score of 0.98, a sensitivity and NPV of 0.97, and an AUC of 1.00. YOLOv4 trained on AP views showed slightly lower performance, with an accuracy and F1-score of 0.96 and an AUC of 1.00. External validation confirmed the strong generalizability of AP-trained models, particularly YOLOv9, which reached an accuracy of 0.87, a sensitivity of 1.00, a specificity of 0.75, a precision of 0.80, an NPV of 1.00, an F1-score of 0.88, and an AUC of 0.93. Artificial intelligence-assisted YOLO models showed strong potential in detecting tibial plateau fractures. **Conclusions:** Models trained on AP views consistently achieved better diagnostic accuracy than those using other views. Among all, YOLOv9 delivered the best results, highlighting the benefits of newer deep learning architectures.

## 1. Introduction

Tibial plateau fractures involve the articular surface of the knee and can be challenging to diagnose due to the complex anatomy of the knee joint and the broad spectrum of fracture patterns [1]. This complexity can lead to some cases being misdiagnosed on plain radiographs [2]. Delayed diagnosis of tibial plateau fractures can result in poor fracture healing and post-traumatic arthritis, causing persistent pain, reduced joint function, and increased healthcare costs [3]. Current diagnostic methods for tibial plateau fractures include X-rays, computed tomography (CT), magnetic resonance imaging (MRI), and bone scans. X-rays are the most commonly used first-line diagnostic tool for clinicians due to their low cost and ease of operation. However, when diagnosing knee fractures, the sensitivity and specificity of X-rays are only 89% and 95%, respectively [4]. This underscores the limited ability of X-rays to detect fractures accurately, particularly in cases involving subtle fracture lines or complex anatomical structures. To improve diagnostic accuracy, artificial intelligence (AI) technology has been applied to assist clinicians in making faster and more precise diagnoses.

AI has a broad range of applications in clinical practice, including disease diagnosis, treatment planning, prognosis prediction, and personalized medicine [5]. The implementation of AI not only enhances diagnostic and therapeutic accuracy but also improves patient satisfaction and decreases the occurrence of medical disputes [6,7]. With the rapid development of medical imaging technology, the volume and complexity of medical imaging data have greatly increased, resulting in a greater demand for specialized personnel, time, and medical costs for interpretation [8]. This has also driven advancements in computer-aided diagnostic technologies in recent years [9]. Among the various object detection algorithms, the YOLO (You Only Look Once) model has become a crucial tool in medical image analysis due to its exceptional efficiency and accuracy [10]. By taking the entire image as input and performing object localization and classification in a single forward propagation, it significantly enhances detection speed. However, the performance of different versions of the YOLO model varies across different application scenarios, indicating that selecting the appropriate model is critical for optimizing diagnostic accuracy and efficiency in specific clinical contexts [11]. Tibial plateau fractures, in particular, are often first evaluated on plain radiographs by non-specialist clinicians in time-pressured emergency settings, where subtle or complex fracture patterns are at risk of being overlooked, creating a clear need for robust real-time decision-support tools.

Previous research has successfully applied AI models to diagnose various orthopedic conditions, including spinal disorders, wrist fractures, and hip fractures [12,13]. However, studies targeting the diagnosis of tibial plateau fractures using AI remain limited [14,15,16]. These studies have demonstrated that deep learning models can assist in detecting tibial plateau fractures, improving sensitivity, supporting occult or complex fracture identification, and in some cases reducing interpretation time for clinicians, thereby underscoring the clinical potential of AI in this domain. Notably, there is still no consensus regarding the optimal radiographic view for training AI models in detecting such fractures. This lack of standardization may affect model accuracy and clinical applicability. Additionally, different AI model architectures offer unique capabilities that can significantly enhance diagnostic performance, particularly in identifying intricate anatomical structures and subtle fracture patterns [7]. The diagnostic performance of various AI models for tibial plateau fractures remains uncertain. The YOLO model, in particular, with its architecture optimized for real-time object detection, has not yet been systematically evaluated for the diagnosis of this specific fracture type. To our knowledge, no prior study has systematically compared multiple generations of YOLO architectures across different radiographic views for tibial plateau fracture detection on plain radiographs, leaving the relative trade-offs between model version and view selection unclear in this setting.

This study aims to investigate the impact of various imaging views in X-rays, such as anteroposterior (AP), lateral, and combined AP and lateral views, on the detection performance of YOLO models. It also compares the performance of different YOLO model versions, specifically YOLOv4, YOLOv5, YOLOv8, and YOLOv9, in detecting tibial plateau fractures. It is hypothesized that models trained with integrated multi-view images and newer YOLO model versions will result in superior performance. Through this study, we seek to identify the most promising model to assist clinicians in accurately identifying tibial plateau fractures, thereby improving diagnostic precision, reducing the risk of misdiagnosis, and ultimately enhancing patient outcomes.

## 2. Background Review and Related Study

### 2.1. YOLO Models

The YOLO series of models represents a groundbreaking advancement in real-time object detection, known for its speed and accuracy [10]. Unlike traditional two-stage detectors, which first generate region proposals and then perform classification, YOLO adopts a single-stage architecture that directly predicts bounding boxes and class probabilities from the entire image in a single forward pass. This unified approach significantly reduces computation time while maintaining competitive accuracy. Each successive version of YOLO incorporates architectural enhancements and innovative techniques to meet the evolving demands of real-time AI applications, including medical imaging, autonomous vehicles, and video surveillance.

Released in April 2020, YOLOv4 marked a significant leap in object detection by combining speed and accuracy enhancements. Designed to run efficiently on a single GPU, it incorporated innovative features such as CSPNet for better gradient propagation, Mish activation for smoother optimization, and Mosaic data augmentation for more robust training [17]. These advancements allowed YOLOv4 to outperform YOLOv3 with a 10% improvement in Average Precision (AP) and a 12% increase in Frames Per Second (FPS) performance on the COCO dataset. Its efficient, single-stage architecture made it widely applicable to real-world use cases requiring high-speed and high-accuracy detection.

YOLOv5, developed by Ultralytics, introduced further enhancements in detection efficiency and ease of deployment. Its architecture emphasized lightweight design and compatibility with modern machine learning workflows. YOLOv5 introduced a flexible framework for training and inference, offering built-in augmentation, pretrained weights, and integration with popular libraries like PyTorch 1.9.0 [18]. While specific architectural details were not published in peer-reviewed journals, its adoption became widespread due to its superior usability and performance in real-world applications, especially in edge computing and resource-constrained environments.

YOLOv8, also by Ultralytics, pushed the boundaries of speed and accuracy in real-time object detection. In addition to object detection, it supports tasks like tracking, instance segmentation, and pose estimation. Its architecture incorporates improvements for hardware acceleration, leveraging technologies such as NVIDIA A100 GPUs with TensorRT at FP16 precision [19]. This allows YOLOv8 to achieve faster inference times while maintaining high mean Average Precision (mAP). Its user-friendly API and simplified deployment make it a preferred choice in fields requiring high-performance AI solutions, such as healthcare diagnostics.

YOLOv9 introduces significant innovations such as the Generalized Efficient Layer Aggregation Network (GELAN) and Programmable Gradient Information (PGI), both of which enhance the efficiency of the model. GELAN improves gradient propagation across layers, while PGI optimizes the use of gradient information, resulting in better training performance. These enhancements not only reduce model size but also decrease computational demands, all while maintaining high detection accuracy. When compared to YOLOv8, YOLOv9 achieves a remarkable 49% reduction in parameters and a 43% reduction in computational cost, offering a 0.6% improvement in mAP on the COCO dataset [20]. The combination of these features makes YOLOv9 particularly well suited for complex tasks that require lightweight, high-precision models, such as medical imaging, where computational efficiency and accuracy are paramount.

### 2.2. Metrics of Model Performance

The models were evaluated based on key performance metrics derived from the confusion matrix, including accuracy, sensitivity, specificity, precision, negative predictive value (*NPV*), and F1-score [21]. The confusion matrix provides a comprehensive overview of the model’s performance by categorizing predictions into four groups: true positives (*TP*), true negatives (*TN*), false positives (*FP*), and false negatives (*FN*). A *TP* was defined as an image where the model correctly identified the presence of a tibial plateau fracture. In contrast, a *TN* referred to images where the model correctly recognized the absence of a fracture. *FP* occurred when the model incorrectly predicted the presence of a fracture in an image where no fracture was present. Conversely, *FN* represented images where the model failed to detect a fracture in an image that did, in fact, contain a tibial plateau fracture. The following formulas were used to calculate the performance metrics:

1. Accuracy: The proportion of correctly classified images (both fractures and non-fractures) among all images.
  (1) $$\mathrm{Accuracy}=\frac{TP+TN}{TP+FP+TN+FN}$$
2. Sensitivity (also known as Recall or True Positive Rate): The ability of the model to correctly identify fractures.
  (2) $$\mathrm{Sensitivity}=\frac{TP}{TP+FN}$$
3. Specificity (also known as True Negative Rate): The ability of the model to correctly identify non-fractures.
  (3) $$\mathrm{Specificity}=\frac{TN}{TN+FP}$$
4. Precision: The proportion of images predicted as fractures that are actual fractures.
  (4) $$\mathrm{Precision}=\frac{TP}{TP+FP}$$
5. NPV: The proportion of images predicted as non-fractures that are actual non-fractures.
  (5) $$\mathrm{NPV}=\frac{TN}{TN+FN}$$
6. F1-score: The harmonic mean of precision and sensitivity, providing a balance between these two metrics.
  (6) $$F1-\mathrm{score}=\frac{2\times \mathrm{Precision}\times \mathrm{Sensitivity}}{\mathrm{Precision}+\mathrm{Sensitivity}}$$

In addition to final test metrics, the training process was monitored using several dynamic performance curves to evaluate model convergence and learning stability. These included the Recall-Confidence Curve (R-curve), Precision-Confidence Curve (P-curve), Precision-Recall Curve (PR- curve), and F1-Confidence Curve (F1-curve). Due to differences in training frameworks, YOLOv4 was implemented using the original Darknet environment, which produced distinct outputs such as loss and mean Average Precision (mAP) curves. In contrast, YOLOv5, YOLOv8, and YOLOv9 were trained using the Ultralytics framework, which generated standardized curves for precision, recall, PR, and F1-score. Model discrimination was further assessed using the Receiver Operating Characteristic (ROC) curve and the corresponding Area Under the Curve (AUC).

## 3. Materials and Methods

### 3.1. Data Sources

Ethical approval for this study was obtained from the Institutional Review Board of Taichung Veterans General Hospital (CE25168C). Knee X-rays were retrospectively collected from cases diagnosed with and without tibial plateau fractures between January 2013 and June 2023. The inclusion criteria for images are as follows: (1) patients aged 18 years or older, (2) X-rays obtained in one of the following imaging techniques: knee supine, knee standing, or knee Rosenberg, and (3) the presence of both AP and lateral views. The exclusion criteria include: (1) images containing implants, (2) significant knee rotation that impairs assessment, (3) associated fractures, (4) poor image quality, and (5) old fractures. All images were reviewed and verified by two orthopedic surgeons to ensure they met the study criteria. In cases where the diagnosis was uncertain, additional imaging modalities such as CT or MRI were referenced to assist in the evaluation. In compliance with privacy regulations, all personal identifiers were removed from the X-ray to ensure patient confidentiality. Knee X-rays were exported from Picture Archiving and Communication System (PACS) (SmartIris 1.3.0.14, Taiwan Electronic Data Processing Corp., Taipei, Taiwan).

### 3.2. Study Design

**Figure 1:**
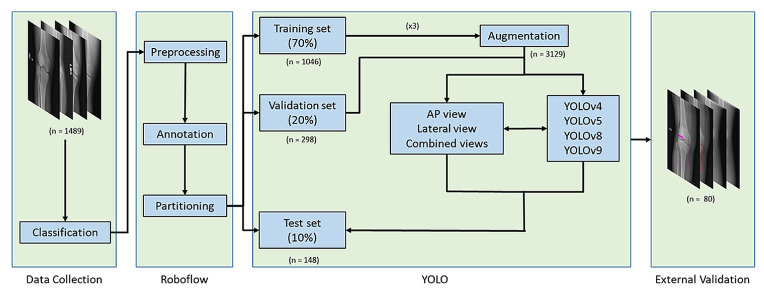
Study Workflow for Model Development and Validation. Knee X-rays were classified, preprocessed, and randomly split into training, validation, and test sets. YOLO models were trained using AP, lateral, and combined views of radiographs with data augmentation. External validation was performed on a dataset from a separate regional hospital.

The images were first classified into four datasets based on AP or lateral views and fracture or non-fracture cases. The dataset’s preprocessing, annotation, partitioning, and augmentation were performed using the online Roboflow computer vision tools (https://universe.roboflow.com/). Initially, all images were resized to 640×640 pixels and annotated as either fracture or non-fracture. The dataset was then divided into training (70%), validation (20%), and test sets (10%) to ensure a balanced evaluation. To enhance data diversity and improve model robustness, data augmentation techniques are applied to the training set. This augmented training set is then used to train three YOLO model variants, including YOLOv4, YOLOv5, YOLOv8, and YOLOv9, under consistent parameter settings to facilitate fair comparison. Each model was trained separately on AP view, lateral view, and combined AP and lateral views. The validation set aids in tuning model parameters, preventing overfitting and improving adaptability to various imaging conditions. The test set, kept entirely separate from the training process, is employed for final model evaluation. Model performance was assessed using multiple metrics to ensure a comprehensive analysis, and training convergence was monitored using performance curves to evaluate model stability. Finally, an additional external validation dataset consisting of 40 fracture images and 40 non-fracture images was collected from another regional hospital using the same inclusion and exclusion criteria. These images were acquired with knee radiography protocols consistent with those of the primary site to evaluate the model’s clinical applicability across institutions (Figure 1).

### 3.3. Data Annotation

**Figure 2:**
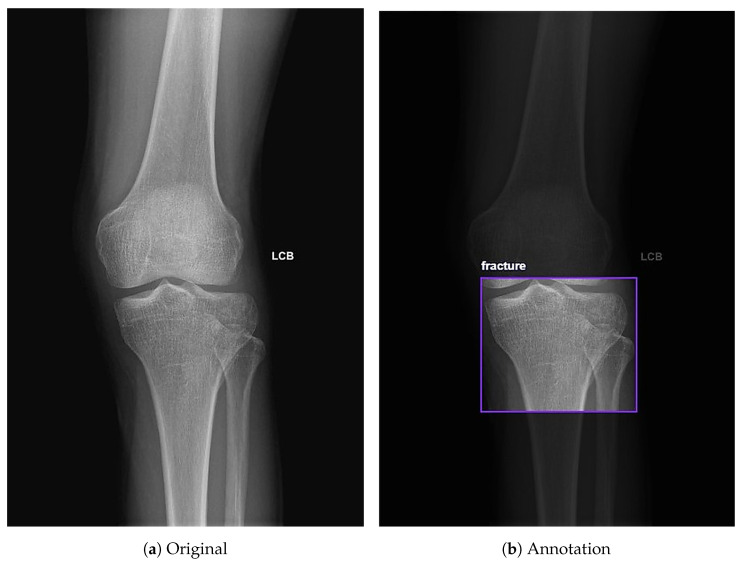
Annotation of knee X-rays using Roboflow. (**a**) Full-field AP view of the knee. (**b**) Example of an annotation on the tibial plateau labeled as fracture using Roboflow.

The online Roboflow computer vision tools were used to classify and annotate knee X-rays. Radiographs included standard clinical orientation markers, such as LCB (Left-Corrected-Beam), which indicate laterality. Each image was manually annotated with bounding boxes centered on the proximal tibia region, allowing the model to focus on and learn features from these specific areas. For each radiograph, a single bounding box was drawn to encompass the proximal tibial articular surface and labeled as either “fracture” or “non-fracture”, even when multiple fracture lines or depressed regions were present, to mirror the clinical emphasis on determining whether a tibial plateau fracture is present rather than exhaustively delineating every individual fracture line. Specifically, the dataset was labeled into two categories: fracture cases, labeled as “fracture”, and non fracture cases, labeled as “non-fracture” (Figure 2). Additionally, Roboflow automated the generation of the YOLO-compatible text files required for model training and evaluation.

### 3.4. Data Partitions and Augmentation

Each image in the dataset was assigned a unique identifier and then randomly partitioned at the image level into training, validation, and test sets with a 70%, 20%, and 10% distribution, respectively. To improve the robustness and generalizability of the model across varied imaging conditions, data augmentation was performed on the training dataset. This included flipping images horizontally, rotation adjustments ranging from $-10^{\circ }\;\mathrm{to}+\;10^{\circ }$, brightness adjustments ranging from −10%to+10%, and exposure correction varying from −10%to+10%. These augmentation methods expanded the original dataset threefold.

### 3.5. Models and System Setup

YOLOv4, YOLOv5, YOLOv8, and YOLOv9 were selected as the primary models for training, using mostly consistent parameters to facilitate fair comparison. The standard configuration included an image size of 640 × 640 pixels, batch size of 16, and 300 training epochs. Two exceptions were applied: for YOLOv9, the batch size was reduced to 8 due to hardware constraints; for YOLOv4, the number of epochs was increased to 4000 based on the recommended formula (epochs = number of classes × 2000), given the binary classification task.

### 3.6. System Configuration

The training was conducted in a hardware and software environment specifically configured to optimize performance and efficiency for deep learning algorithms. This setup was essential for handling extensive image processing and achieving fast inference times during model training. A detailed description of the system configuration is provided in Table 1.

## 4. Results

### 4.1. Patient Demographics and Image Counts Before and After Augmentation

A total of 677 cases were included, consisting of 397 females and 280 males. The mean age of the population was 41.32 ± 18.19 years. The initial dataset comprised 1489 knee X-rays, including 727 fracture images and 762 non-fracture images. Specifically, there were 363 AP and 364 lateral view images of fractures, along with 383 AP and 379 lateral view images of non-fractures. Following data augmentation applied to the training set, the dataset expanded to 3575 images. Detailed counts of images in each dataset, both before and after augmentation, are presented in Table 2.

### 4.2. Model Training Process

**Figure 3:**
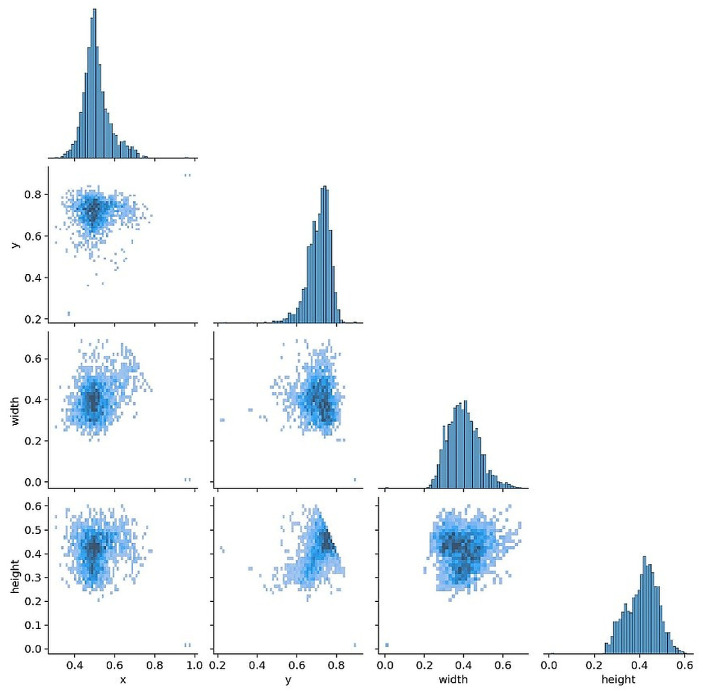
Labels correlogram. Pairwise distributions of normalized box variables (x, y, width, height). Centers cluster near x about 0.50 and y 0.60 to 0.65, with a moderate positive correlation between width and height.

**Figure 4:**
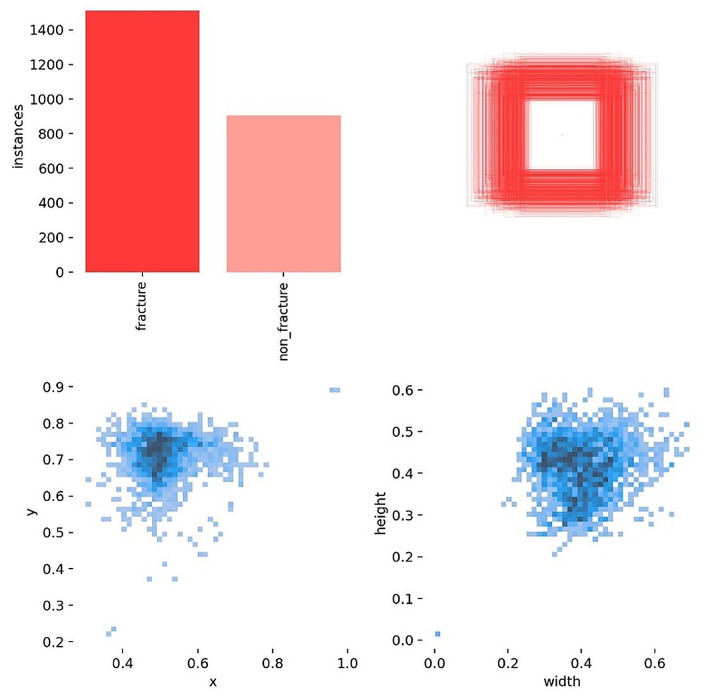
Labels distributed. Class counts are near balanced; box centers concentrate over the proximal tibia, and scales and aspect ratios are consistent across images.

Before training, label and box diagnostics were conducted to verify annotation quality and to guide anchor and augmentation choices. The correlogram (Figure 3) shows pairwise relationships among normalized box variables (x, y, width, height): centers cluster near x about 0.50 and y around 0.60 to 0.65, width and height are moderately positively correlated, and box size is largely independent of horizontal position. The distribution view (Figure 4) confirms near balanced class counts (fracture and non-fracture), tight concentration of box centers over the proximal tibia, and consistent scales and aspect ratios across images.

**Figure 5:**
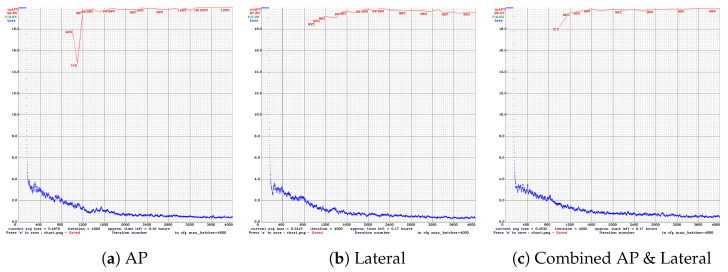
Training performance of YOLOv4 across different radiographic views. (**a**) AP view shows early mAP fluctuations but good convergence. (**b**) Lateral view has slower convergence and lower mAP. (**c**) Combined views achieve stable and high mAP.

The following figures summarize the training behavior and performance metrics of each YOLO model across different imaging views, providing a comparative assessment of model convergence, stability, and classification performance. Figure 5 presents the training performance of YOLOv4 under different image views. In the AP view (Figure 5a), the mAP exhibits noticeable fluctuations between iterations 800 and 1200 but subsequently stabilizes at 99.9%, indicating effective convergence. In the lateral view (Figure 5b), the mAP is slightly reduced to 97.8%, accompanied by a slower convergence trend. When trained on combined AP and lateral views (Figure 5c), the model achieves a consistently high mAP of up to 99.4% with minimal variation, demonstrating enhanced learning stability and robustness through multi-view integration.

**Figure 6:**
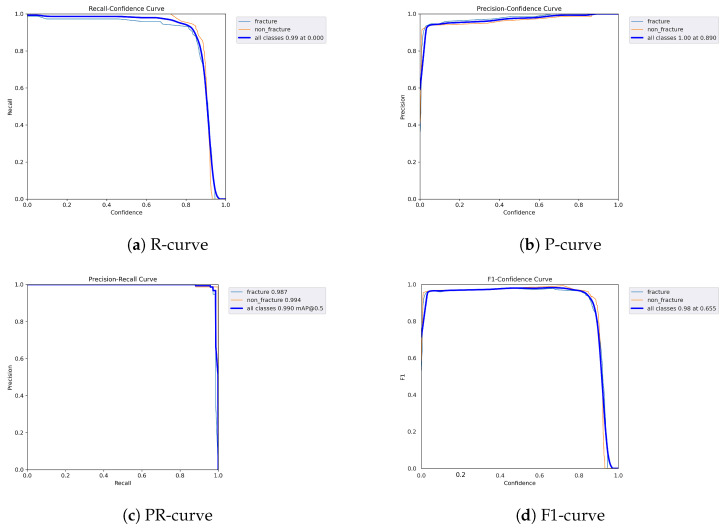
Dynamic performance curves for YOLOv5 (AP view). (**a**) R-curve shows a drop beyond threshold 0.6. (**b**) P-curve remains stable above 0.9. (**c**) PR-curve demonstrates strong area coverage. (**d**) F1-curve shows optimal balance between 0.4 and 0.8.

**Figure 7:**
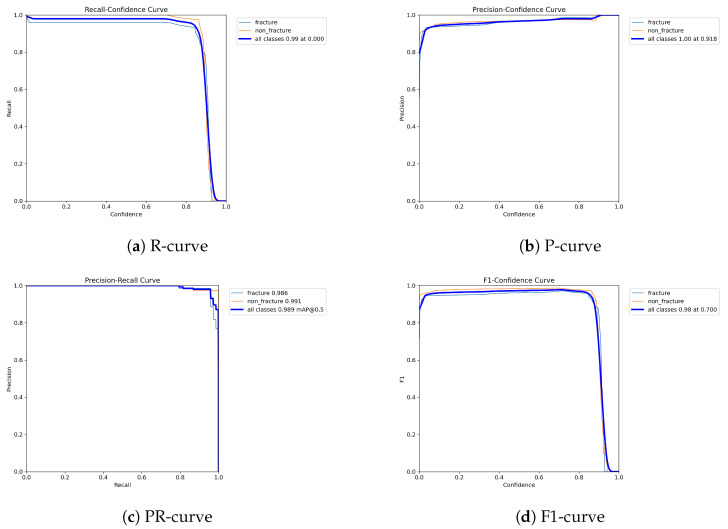
Dynamic performance curves for YOLOv8 (AP view). (**a**) R-curve is smoother than YOLOv5. (**b**) P-curve remains above 0.9. (**c**) PR-curve suggests high sensitivity. (**d**) F1-curve shows a wider plateau of optimal values.

**Figure 8:**
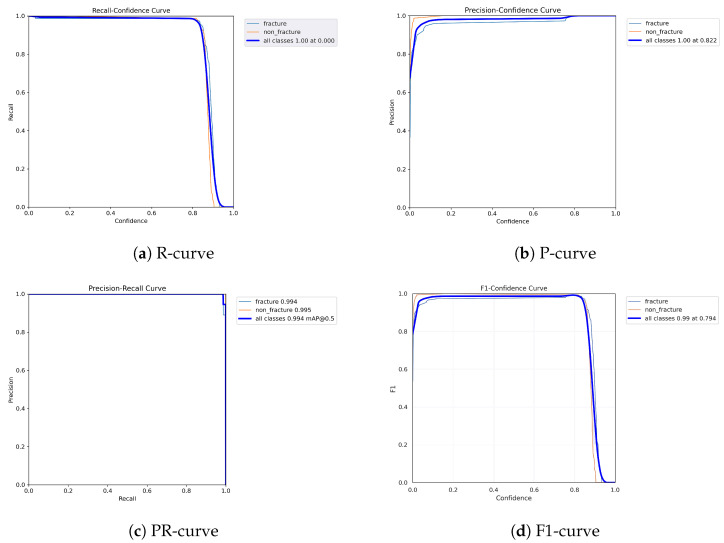
Dynamic performance curves for YOLOv9 (AP view). (**a**) R-curve remains high until 0.8. (**b**) P-curve exceeds 0.9 consistently. (**c**) PR-curve is nearly ideal. (**d**) F1-curve is highly stable across thresholds.

**Figure 9:**
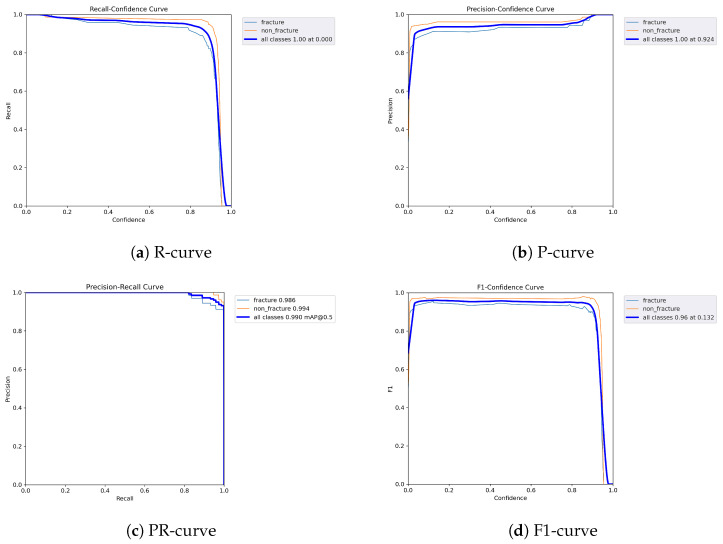
Dynamic performance curves for YOLOv5 (Lateral view). (**a**) R-curve drops earlier than in AP view. (**b**) P-curve is less consistent above 0.7. (**c**) PR-curve shows a moderate decrease in recall. (**d**) F1-curve reflects reduced stability.

**Figure 10:**
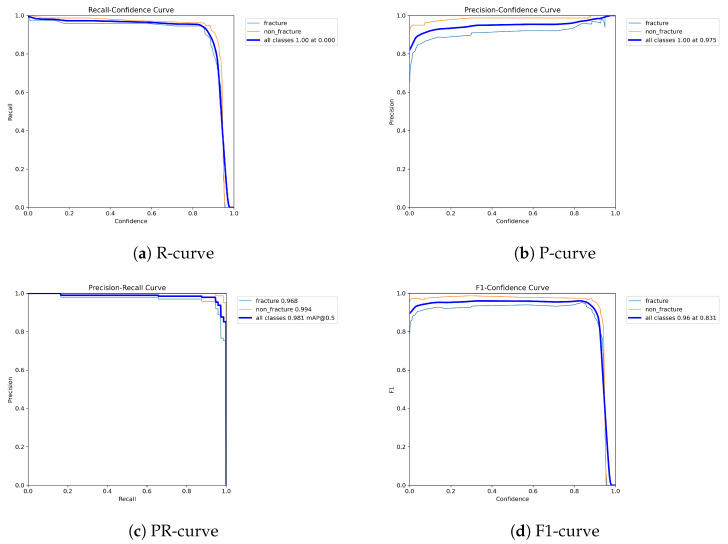
Dynamic performance curves for YOLOv8 (Lateral view). (**a**) R-curve remains relatively high up to 0.8. (**b**) P-curve is steady above 0.85. (**c**) PR-curve displays strong area. (**d**) F1-curve indicates good balance.

**Figure 11:**
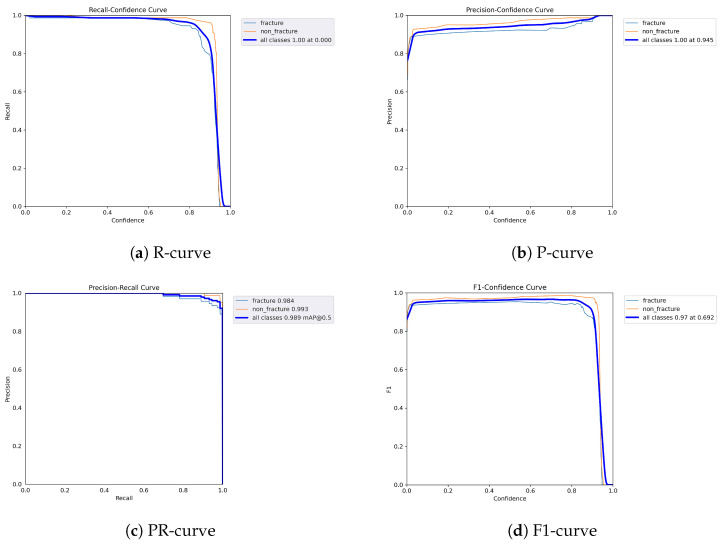
Dynamic performance curves for YOLOv9 (Lateral view). (**a**) R-curve maintains better levels than other models. (**b**) P-curve stays over 0.9. (**c**) PR-curve shows minimal loss. (**d**) F1-curve demonstrates strong robustness.

**Figure 12:**
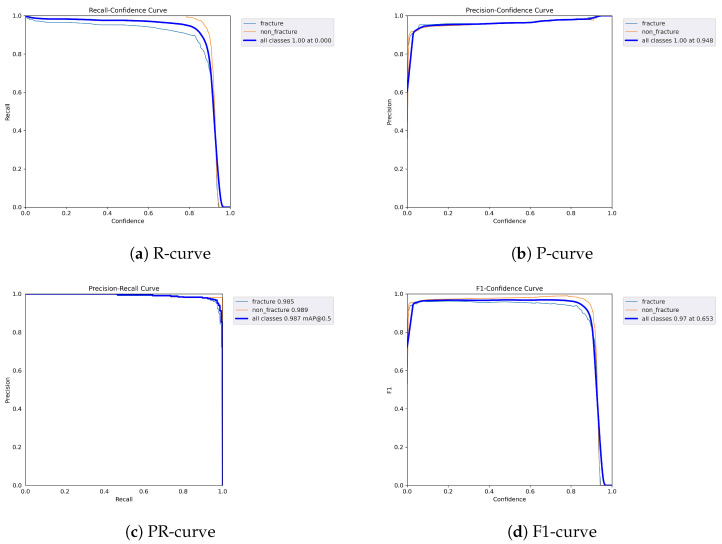
Dynamic performance curves for YOLOv5 (Combined AP & Lateral). (**a**) R-curve declines earlier than single views. (**b**) P-curve remains relatively stable. (**c**) PR-curve is flatter than AP view. (**d**) F1-curve indicates acceptable but variable stability.

**Figure 13:**
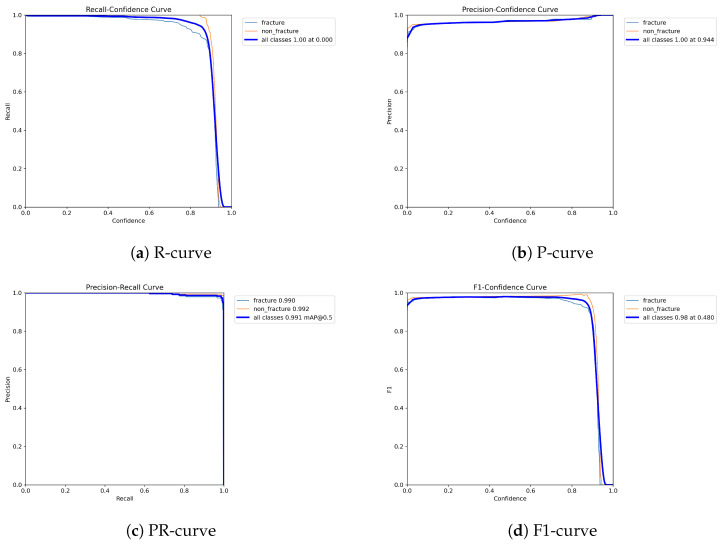
Dynamic performance curves for YOLOv8 (Combined AP & Lateral). (**a**) R-curve is smooth up to 0.8. (**b**) P-curve holds above 0.9. (**c**) PR-curve shows strong generalization. (**d**) F1-curve remains steady across thresholds.

**Figure 14:**
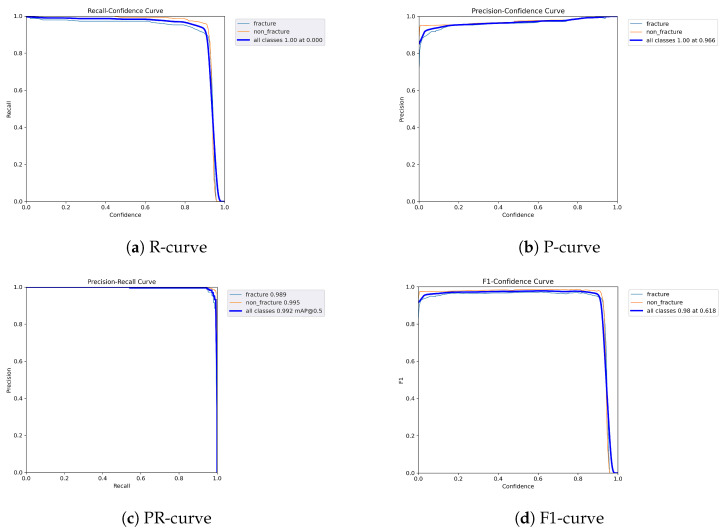
Dynamic performance curves for YOLOv9 (Combined AP & Lateral. (**a**) R-curve sustains high levels throughout. (**b**) P-curve is flat and exceeds 0.9. (**c**) PR-curve closely approximates ideal. (**d**) F1-curve is stable with minimal fluctuations.

The following graphs show dynamic performance curves for YOLOv5 in Figure 6, YOLOv8 in Figure 7, and YOLOv9 in Figure 8 models used to detect fractures in AP view X-rays. Each of the four graphs illustrates a different aspect of the model’s performance as the confidence threshold is varied. In the AP view, YOLOv9 demonstrated superior overall performance, with higher F1-score, precision, and recall compared to YOLOv5 and YOLOv8. Its performance was particularly strong in the low confidence threshold range (0.2–0.5), making it well suited for high-sensitivity fracture detection. While YOLOv8 showed slight improvement over YOLOv5, the difference was mainly observed in its greater stability at mid to high thresholds (0.6–0.8). In the lateral views shown in Figure 9, Figure 10 and Figure 11, all models exhibited a decline in performance; however, YOLOv9 maintained the highest stability, with superior F1-score and recall compared to YOLOv5 and YOLOv8. This suggests a stronger ability to adapt to non-standard imaging angles. In contrast, YOLOv5 and YOLOv8 showed less consistent performance in the lateral view, particularly with a notable drop in recall, which may increase the risk of missed diagnoses. In the analysis combining AP and lateral views shown in Figure 12, Figure 13 and Figure 14, YOLOv9 demonstrated the strongest generalization capability, with a PR curve close to the ideal and minimal fluctuation in F1-score, confirming its suitability for complex clinical scenarios involving varied X-ray angles. In contrast, YOLOv5 and YOLOv8 showed less stable performance under combined views, especially with greater variability in recall, which may impact diagnostic consistency.

### 4.3. Model Performance Across Different Views

Model performance across different views and YOLO versions is summarized in Table 3. Across all YOLO models, the AP view generally demonstrated superior or comparable performance across multiple evaluation metrics. For YOLOv4, the AP view achieved the highest or equal highest values for accuracy (0.96), sensitivity (0.96), specificity (0.97), precision (0.97), NPV (0.96), and F1-score (0.96), all outperforming the lateral view. YOLOv5 showed its best results under the AP view, with the highest accuracy (0.98), sensitivity (0.97), specificity (1.00), precision (1.00), NPV (0.97), and F1-score (0.98), exceeding both lateral and combined views. YOLOv8 also performed best in the AP view, reaching top values in accuracy (0.97), sensitivity (0.97), NPV (0.97), and F1-score (0.97), which were equal to or slightly better than those in the other views. For YOLOv9, although it achieved strong and balanced results across all three views, the AP view yielded the highest values in accuracy (0.99), sensitivity (1.00), specificity (0.99), precision (0.99), NPV (1.00), and F1-score (0.99). These findings suggest that AP-view radiographs provide more consistent and informative fracture features, enabling improved model learning and contributing to enhanced diagnostic performance across different YOLO architectures.

### 4.4. Model Performance Across YOLO Versions

In terms of model comparison, YOLOv9 outperformed the other versions across most evaluation metrics (Table 3). It achieved the highest accuracy (0.99), sensitivity (1.00), NPV (1.00), and F1-score (0.99) under the AP view. Even in the lateral and combined AP & lateral views, YOLOv9 consistently demonstrated high performance across most metrics. In the lateral view, it achieved perfect or near-perfect values, including 0.99 in accuracy, sensitivity, specificity, precision, NPV, and F1-score. Similarly, in the combined view, YOLOv9 maintained strong results with an accuracy of 0.97, sensitivity of 0.96, specificity of 0.99, precision of 0.98, NPV of 0.96, and an F1-score of 0.97. YOLOv8 achieved the highest specificity and precision (both 1.00) in the lateral view, while also maintaining strong performance across other metrics, demonstrating its consistent and reliable detection capability. YOLOv5 showed solid classification results, with a perfect specificity and precision of 1.00 in the AP view. In contrast, YOLOv4 yielded lower performance across all views. In the lateral view, it had the lowest recorded values in this study: accuracy (0.92), sensitivity (0.92), specificity (0.93), Precision (0.93), NPV (0.92), and F1-score (0.92). These results highlight YOLOv9’s superior robustness and discriminative ability, making it the most effective model for tibial plateau fracture detection among the compared versions.

### 4.5. ROC Curve and AUC Analysis

**Figure 15:**
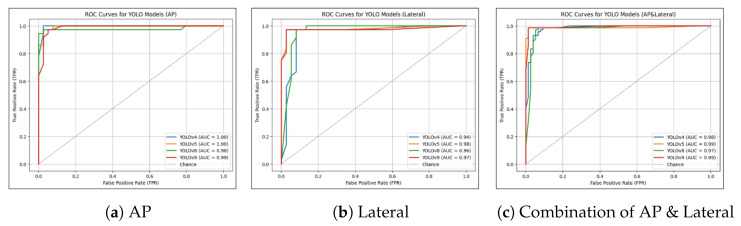
Curves for Different Views and YOLO Versions. (**a**) ROC curves for models trained on the AP view, with YOLOv4 showing the highest AUC of 1.00. (**b**) ROC curves for models trained on the lateral view, with YOLOv5 achieving the highest AUC of 0.98. (**c**) ROC curves for models trained on combined AP and lateral views, with YOLOv9 again exhibiting the best performance with an AUC of 0.99.

The ROC curves and AUC values of the models are shown in Figure 15. All models, regardless of the version or view, achieved AUC values above 0.94. Models trained on the AP view achieved the highest AUCs, ranging from 0.98 to 1.00, compared to those trained on other views. Specifically, models trained on lateral views had AUCs ranging from 0.94 to 0.98, while models trained on combined AP and lateral views ranged from 0.97 to 0.99.

### 4.6. External Validation

**Figure 16:**
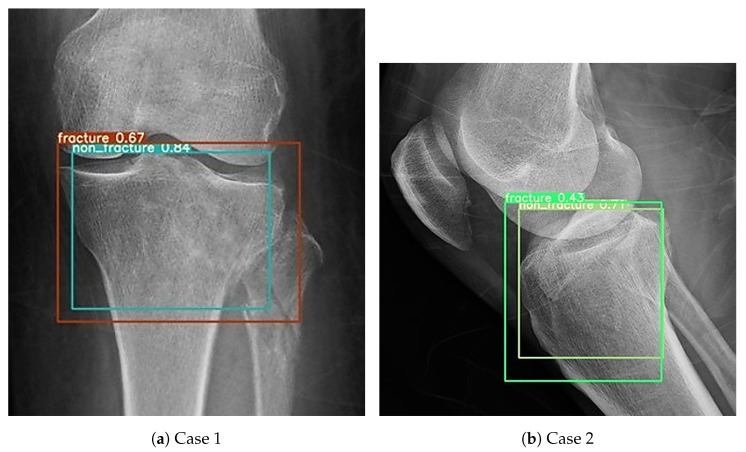
Cases in which both fracture and non-fracture features were detected simultaneously. (**a**) Example where the AP view model detected both fracture and non-fracture features in the same image. (**b**) Example where the lateral view model detected both fracture and non-fracture features in the same image.

During the external validation process, one notable issue was identified. The same image could occasionally be recognized as containing both fracture and non-fracture features, as shown in Figure 16. A total of 18 such images were recorded across all YOLO models. Among them, YOLOv4 was responsible for the majority, with 3 occurrences in the AP view and 7 in the lateral view. YOLOv5 had 2 such images, all from the AP view, while no conflicts were observed in the lateral view. YOLOv8 and YOLOv9 each showed 3 images, with 2 detected in the AP view and 1 in the lateral view. This created a classification challenge. To resolve this, a decision rule was implemented whereby a positive detection in any single view would result in the case being classified as a fracture. This conservative rule was applied consistently across all models and datasets and was chosen to prioritize high sensitivity and negative predictive value, accepting a reduction in specificity and positive predictive value, in order to reflect the clinical preference for avoiding missed fractures. In clinical settings, physicians often prefer to overdiagnose rather than risk overlooking a fracture. Therefore, this classification method was selected to align with clinical priorities. Whenever a fracture feature was detected, the image was classified as a fracture, even if non-fracture features were also present.

External validation confirmed a clear hierarchy among the three radiographic views. Models trained on AP images showed the strongest generalisability, with accuracy ranging from 0.80 to 0.92, F1 scores from 0.77 to 0.93, and AUC values between 0.88 and 0.93 (Table 4). When the same architectures were tested on lateral images, performance declined: accuracy fell to 0.65 to 0.77, F1 scores to 0.72 to 0.80, and AUC to 0.69 to 0.83. Training on both AP and lateral views produced intermediate metrics, with accuracy 0.70 to 0.83, F1 scores 0.69 to 0.85, and AUC 0.79 to 0.89. Across YOLO versions, newer models held an advantage; YOLOv8 and YOLOv9 formed the top tier with accuracy 0.87 to 0.92, F1 scores 0.88 to 0.93, and AUC 0.92 to 0.93, whereas YOLOv4 consistently trailed and YOLOv5 occupied a middle ground.

## 5. Discussion

The results indicate that models trained using AP views generally demonstrated superior performance across various evaluation metrics compared to those trained on lateral or combined views. This finding contrasts with our initial hypothesis. In clinical practice, integrating multiple views is generally considered to provide a more comprehensive representation of tibial plateau fractures by capturing anatomical details that may not be visible in a single projection. Because X-ray imaging inherently compresses three-dimensional structures into two-dimensional projections, the visibility of a fracture line strongly depends on its orientation relative to the X-ray beam. For example, a fracture that lies predominantly in the sagittal plane may be almost invisible on a coronal (AP) projection but becomes much clearer on a lateral view, which explains why subtle or obscured fractures in one view can appear more evident in a complementary perspective. However, combining AP and lateral radiographs within a single detector may also introduce substantial feature heterogeneity and increased image variability, because the two projections emphasize different anatomical structures and contrast patterns. Under our current region-level annotation scheme, where the proximal tibia is labeled as fracture or non-fracture rather than each individual fracture line, these heterogeneous features may not be fused optimally, which could dilute the discriminative information from AP images and partly explain why combined-view models underperformed single-view AP models. A possible explanation is that AP views better visualize key features such as depression or fissures on the medial and lateral articular surfaces, which are critical for fracture identification. Moreover, the most widely used clinical classification system for tibial plateau fractures, the Schatzker classification, is based primarily on AP radiographs, further supporting the clinical relevance of using AP views for accurate fracture characterization [1]. Furthermore, newer YOLO versions demonstrated stronger performance compared to older models, largely due to advancements in model architecture and optimization. In particular, YOLOv9 incorporates more effective feature extraction and fusion mechanisms, along with improved gradient propagation, which enhance its ability to capture subtle cortical irregularities, articular surface depression, and fine fracture lines that characterize tibial plateau injuries. Innovations in the latest versions, such as improved gradient propagation, enhanced feature aggregation, and more efficient use of computational resources, allow for better detection of subtle or intricate fracture characteristics. These architectural and optimization updates not only improve accuracy, sensitivity, and specificity but also help maintain fast training and inference, making newer YOLO models, especially YOLOv9, more suitable for real-time or near–real-time clinical applications.

The clinical significance of this study appears to be considerable. Tibial plateau fractures are typically diagnosed by emergency personnel rather than orthopedic specialists, and the high-pressure environment in busy emergency departments increases the risk of misdiagnosis or missed diagnoses [9,22]. This can lead to delayed or inappropriate treatment, adversely affecting patient recovery [10,23]. Previous studies have shown that X-ray imaging has a sensitivity of 0.89, specificity of 0.95, and both positive predictive value and NPV of 0.92 compared to CT scans for detecting knee fractures, underscoring its diagnostic limitations [4]. Consistently, prior literature has reported that even experienced clinicians may miss or misinterpret traumatic fractures on plain radiographs in emergency settings, further highlighting the challenges of relying solely on human readers in this context [2]. In contrast, our study found that AI-assisted YOLOv9 analysis on AP views achieved high sensitivity, specificity, precision, and NPV, reflecting high internal accuracy and moderate external performance that may complement conventional radiographic interpretation. However, this remains an indirect comparison, and a prospective reader study directly evaluating clinicians with and without AI assistance will be essential to establish the true clinical impact and practical utility of the proposed system. Implementing AI and machine learning technologies can significantly reduce misdiagnosis rates, benefiting both the healthcare system and patients. Particularly in areas with limited resources or a lack of specialist availability, such technology can provide diagnostic support, addressing gaps in medical resources. Additionally, AI technologies can effectively lower healthcare costs by reducing unnecessary tests and treatments, shortening hospital stays, and minimizing additional expenses due to disease progression, thus controlling overall medical expenditures and enhancing healthcare efficiency [24]. For medical training and education, AI models can offer diverse and accurate cases, aiding in the accumulation of experience during the learning phase, enhancing diagnostic skills and clinical decision-making, and further improving the quality of medical services [25].

In the existing literature, research focused on the application of AI in tibial plateau fractures remains limited [14,15,16,26,27]. Liu et al. demonstrated that a RetinaNet-based deep learning model performed well in detecting tibial plateau fractures on AP view of the knee [14]. The study showed that the AI model achieved an accuracy of 0.91, closely matching the 0.92 accuracy of orthopedic physicians. Van der Gaast et al. applied the GoogleNet algorithm using both AP and lateral knee radiographs for fracture detection, reporting high sensitivity (92.7%) but only moderate accuracy (70.4%) and positive predictive value (64.4%), indicating strong capability in identifying fractures but with a higher false positive rate [15]. More recently, Huo et al. reported a multicenter deep learning system for adult tibial plateau fractures, demonstrating that AI assistance can improve sensitivity and efficiency, especially for less experienced readers [16]. Taken together, these studies suggest that deep learning can augment tibial plateau fracture diagnosis but mainly rely on classification frameworks and offer limited comparison across detector types or radiographic views. To our knowledge, this is the first study to employ the YOLO architecture, a real-time, single-stage object detection model, in the analysis of tibial plateau fractures. Although direct comparisons between YOLO and other architectures were not conducted in this study, the performance achieved by our YOLOv9 model trained on AP images represents the highest reported to date in this domain, with sensitivity and accuracy reaching 1.00 and 0.99, respectively. These findings highlight the potential of advanced object detection models in improving diagnostic accuracy and efficiency in musculoskeletal imaging. Additionally, our study offers several improvements over previous research. First, our study explored the diagnostic performance across different radiographic views, including AP, lateral, and combined views, aiming to determine the optimal perspective for AI-assisted fracture detection. Although clinical consensus suggests that combining multiple views enhances diagnostic accuracy, prior literature has not clearly demonstrated the comparative value of each view [28]. Our findings address this gap by systematically analyzing performance variations across views and demonstrating that models trained solely on AP views often outperformed those trained on lateral or combined views. We also conducted extensive training and testing across different YOLO models and selected the most clinically useful model based on these evaluations, ensuring more reliable and practical diagnostic outcomes.

This study employs an object detection framework but is operationally formulated as a region-level binary decision task, in which the model predicts the presence or absence of a tibial plateau fracture within a single proximal tibia region per image; multiple fracture lines or separate fracture areas within this region are treated as a single positive event rather than distinct lesions. AI technology shows great potential in the application of tibial plateau fractures, not only assisting in the detection of fractures but also identifying associated injuries and aiding in disease classification [26,27,29]. Beyond traditional X-rays, AI applications in other imaging modalities such as CT and MRI are increasingly being explored, offering valuable insights for complex case management. Xie et al. demonstrated that deep learning applied to MRI images can accurately analyze tibial plateau fractures combined with meniscus injuries [26]. Complex knee joint injuries often accompany tibial plateau fractures, and MRI provides more detailed soft tissue imaging, allowing AI models to accurately detect and diagnose these injuries. Furthermore, AI has shown great potential in the classification of tibial plateau fractures. Lind et al. demonstrated the use of AI to assist in applying the AO/OTA classification system for tibial plateau fracture classification [29]. Meanwhile, Cai et al. showcased the ability of three-dimensional U-Net in automatically segmenting CT images and assisting physicians with Schatzker classification [27]. The application of AI not only accelerates the classification process but also enhances its accuracy, providing valuable support for preoperative planning and treatment decisions.

This study has limitations. First, the use of retrospective data from a limited number of institutions may introduce selection bias, as the data may not capture the full diversity of cases seen in different clinical settings, limiting the model’s generalizability. Moreover, potential domain shifts between institutions, such as differences in digital radiography systems, image post-processing, and patient populations, may have contributed to the performance drop observed in external validation, and this heterogeneity could further affect the robustness of the model across broader clinical environments. No explicit cross-center image normalization or harmonization procedures were applied, which may further accentuate these inter-institutional differences. Additionally, the lack of standardized image quality in retrospective data could impact the AI model’s accuracy and reliability. Furthermore, although data augmentation was applied to increase image variability and improve generalizability, a direct comparison between models trained with and without augmentation was not performed, and this omission should be regarded as a limitation that warrants systematic evaluation in future work. In addition, images with metallic implants or severe rotational malposition were excluded during dataset construction to ensure labeling clarity, which may introduce spectrum bias and limit the model’s applicability to more challenging real-world radiographs. Second, the dataset split was performed at the image level rather than strictly at the patient level, which may introduce a risk of optimistic performance estimates because radiographs from the same patient could appear in different subsets; therefore, the reported metrics should be interpreted as upper-bound estimates, and future studies should adopt patient-level splitting and multi-center validation. Third, the study did not include a direct comparison between the AI model’s performance and medical experts during the testing phase. This omission limits the ability to assess whether the AI model can match or surpass human diagnostic accuracy in real-world clinical settings. Fourth, this study did not extend beyond the basic classification of tibial plateau fractures to include further disease categorization. The absence of detailed fracture type or severity classification limits the model’s ability to provide nuanced diagnostic insights, which could be crucial for tailored treatment planning. Fifth, the study did not compare the YOLO models with other algorithms commonly used in medical image analysis, nor did it include more recent YOLO iterations beyond YOLOv9. Such omissions limit the breadth of the comparative evaluation and the ability to fully characterize the relative strengths and weaknesses of the proposed approach. Future work should therefore incorporate both alternative state-of-the-art detection or classification frameworks and newer YOLO versions to provide a more comprehensive performance benchmark.

## 6. Conclusions

This study significantly advances the understanding of AI applications in detecting tibial plateau fractures on radiographic images. It makes three main contributions to the existing literature: (1) a systematic analysis of radiographic views (AP, lateral, and combined), (2) a comprehensive comparison of multiple YOLO model versions, and (3) the first application of the YOLO architecture specifically to tibial plateau fractures. This study suggests that YOLO models trained on AP views may perform better than those trained on lateral or combined views. Newer YOLO versions generally outperformed older models. Among the models tested, YOLOv9 trained on AP views demonstrated the highest accuracy, specificity, precision, F1-score, and AUC of 0.99, along with sensitivity and NPV of 1.00, showing promise in assisting with radiographic interpretation. These findings indicate that AI could serve as a supportive tool in musculoskeletal diagnostics, potentially improving accuracy and efficiency in fracture detection.

## Figures and Tables

**Table 1:** System Configuration for Model Training.

Configuration/Component	Specification
Operating System	Ubuntu 22.04
Hardware Configuration	
CPU	Intel Xeon Gold 6226R
GPU	NVIDIA RTX 8000P-12Q
Software Configuration	
CUDA	Version 12.2
cuDNN	Version 8.9.4.25
CMake	Version 3.22.1
Python	Version 3.10.12
OpenCV	Version 4.5.4
NumPy	Version 1.23.5

**Table 2:** Image Numbers in the Dataset Before and After Augmentation.

Category	View	Train Set		Valid Set	Test Set	Total	
		Original	Augmented			Original	Augmented
Fracture	AP	254	762	73	36	363	871
	Lateral	255	765	73	36	364	874
	Subtotal	509	1527	146	72	727	1745
Non-fracture	AP	268	804	76	39	383	919
	Lateral	266	798	76	37	379	911
	Subtotal	534	1602	152	76	762	1830
Grand Total		1046	3129	298	148	1489	3575

**Table 3:** Model Performance Across Different Views and YOLO Versions.

Model	View	Duration (h)	Acc	Sens	Spec	Prec	NPV	F1	AUC
**YOLOv4**	AP	5.50	0.96	0.96	0.97	0.97	0.96	0.96	**1.00**
	Lateral	5.68	0.92	0.92	0.93	0.93	0.92	0.92	0.94
	AP & Lateral	5.46	0.96	0.96	0.96	0.95	0.96	0.96	0.98
**YOLOv5**	AP	1.34	0.98	0.97	**1.00**	**1.00**	0.97	0.98	**1.00**
	Lateral	2.55	0.95	0.94	0.97	0.96	0.94	0.95	0.98
	AP & Lateral	2.45	0.97	0.95	0.97	0.98	0.95	0.96	0.99
**YOLOv8**	AP	1.23	0.97	0.97	0.97	0.97	0.97	0.97	0.98
	Lateral	2.07	0.96	0.92	**1.00**	**1.00**	0.92	0.95	0.96
	AP & Lateral	2.58	0.97	0.96	0.99	0.98	0.96	0.97	0.97
**YOLOv9**	AP	4.20	**0.99**	**1.00**	0.99	0.99	**1.00**	**0.99**	0.99
	Lateral	5.34	**0.99**	0.99	0.99	0.99	0.99	**0.99**	0.97
	AP & Lateral	10.13	0.97	0.96	0.99	0.98	0.96	0.97	0.99

**Table 4:** External Validation Results of YOLO Models Across Different Views.

Model	View	Acc	Sens	Spec	Prec	NPV	F1	AUC
	AP	0.80	0.70	0.90	0.87	0.75	0.77	0.88
**YOLOv4**	Lateral	0.75	0.70	0.80	0.77	0.72	0.73	0.83
	AP & Lateral	0.76	0.55	**0.97**	**0.95**	0.68	0.69	0.79
	AP	0.85	**1.00**	0.70	0.76	**1.00**	0.86	0.90
**YOLOv5**	Lateral	0.75	**1.00**	0.50	0.66	**1.00**	0.80	0.78
	AP & Lateral	0.83	0.97	0.70	0.76	0.96	0.85	0.89
	AP	**0.92**	**1.00**	0.85	0.86	**1.00**	**0.93**	0.92
**YOLOv8**	Lateral	0.77	0.95	0.60	0.70	0.92	0.80	0.69
	AP & Lateral	0.78	0.97	0.60	0.70	0.96	0.82	0.81
	AP	0.87	**1.00**	0.75	0.80	**1.00**	0.88	**0.93**
**YOLOv9**	Lateral	0.65	0.90	0.40	0.60	0.80	0.72	0.78
	AP & Lateral	0.70	0.97	0.42	0.62	0.94	0.76	0.83

## Data Availability

The data presented in this study are available on request from the corresponding author due to the secure environment of our institution following appropriate review and approval.

## References

[1] Kfuri M., Schatzker J. (2018). Revisiting the Schatzker classification of tibial plateau fractures. Injury.

[2] Pinto A., Berritto D., Russo A., Riccitiello F., Caruso M., Belfiore M.P. (2018). Traumatic fractures in adults: Missed diagnosis on plain radiographs in the Emergency Department. Acta Biomed..

[3] Castano Betancourt M.C., Maia C.R., Munhoz M., Morais C.L., Machado E.G. (2022). A review of Risk Factors for Post-traumatic hip and knee osteoarthritis following musculoskeletal injuries other than anterior cruciate ligament rupture. Orthop. Rev..

[4] Avci M., Kozaci N. (2019). Comparison of X-Ray Imaging and Computed Tomography Scan in the Evaluation of Knee Trauma. Medicina.

[5] Busnatu Ș., Niculescu A.G., Bolocan A., Petrescu G.E., Păduraru D.N., Năstasă I. (2022). Clinical Applications of Artificial Intelligence-An Updated Overview. J. Clin. Med..

[6] Lin H., Li R., Liu Z., Chen J., Yang Y., Chen H., Lin Z., Lai W., Long E., Wu X. (2019). Diagnostic Efficacy and Therapeutic Decision-making Capacity of an Artificial Intelligence Platform for Childhood Cataracts in Eye Clinics: A Multicentre Randomized Controlled Trial. EClinicalMedicine.

[7] Yin J., Ngiam K.Y., Teo H.H. (2021). Role of Artificial Intelligence Applications in Real-Life Clinical Practice: Systematic Review. J. Med. Internet Res..

[8] Smith-Bindman R., Kwan M.L., Marlow E.C., Theis M.K., Bolch W., Cheng S.Y., Bowles E.J., Duncan J.R., Greenlee R.T., Kushi L.H. (2019). Trends in Use of Medical Imaging in US Health Care Systems and in Ontario, Canada, 2000–2016. JAMA.

[9] Al-Antari M.A. (2023). Artificial Intelligence for Medical Diagnostics-Existing and Future AI Technology!. Diagnostics.

[10] Ragab M.G., Abdulkadir S.J., Muneer A., Alqushaibi A., Sumiea E.H., Qureshi R., Al-Selwi S.M., Alhussian H. (2024). A Comprehensive Systematic Review of YOLO for Medical Object Detection (2018 to 2023). IEEE Access.

[11] Catargiu C., Cleju N., Ciocoiu I.B. (2024). A Comparative Performance Evaluation of YOLO-Type Detectors on a New Open Fire and Smoke Dataset. Sensors.

[12] Innocenti B., Radyul Y., Bori E. (2022). The Use of Artificial Intelligence in Orthopedics: Applications and Limitations of Machine Learning in Diagnosis and Prediction. Appl. Sci..

[13] Lisacek-Kiosoglous A.B., Powling A.S., Fontalis A., Gabr A., Mazomenos E., Haddad F.S. (2023). Artificial intelligence in orthopaedic surgery. Bone Jt. Res..

[14] Liu P.R., Zhang J.Y., Xue M.D., Duan Y.Y., Hu J.L., Liu S.X., Xie Y., Wang H.L., Wang J.W., Huo T.T. (2021). Artificial Intelligence to Diagnose Tibial Plateau Fractures: An Intelligent Assistant for Orthopedic Physicians. Curr. Med. Sci..

[15] van der Gaast N., Bagave P., Assink N., Broos S., Jaarsma R.L., Edwards M.J.R., Hermans E., IJpma F.F.A., Ding A.Y., Doornberg J.N. (2025). Deep learning for tibial plateau fracture detection and classification. Knee.

[16] Huo T., Liu P., Xue M., Zhang J., Xie Y., Wang H., Zhou H., Yan Z., Liu S., Lu L. (2025). Deep learning diagnosis of adult tibial plateau fractures: Multicenter study with external validation. Radiol. Adv..

[17] Bochkovskiy A., Wang C.Y., Liao H.Y.M. (2020). YOLOv4: Optimal speed and accuracy of object detection. arXiv.

[18] Khanam R., Hussain M. (2024). What is YOLOv5: A deep look into the internal features of the popular object detector. arXiv.

[19] Wang H., Li D., Isshiki T. (2024). Energy-efficient implementation of YOLOv8, instance segmentation, and pose detection on RISC-V SoC. IEEE Access.

[20] Wang C.-Y., Yeh I.-H., Liao H.-Y. (2024). YOLOv9: Learning What You Want to Learn Using Programmable Gradient Information. arXiv.

[21] Müller A.C., Guido S. (2016). Introduction to Machine Learning with Python: A Guide for Data Scientists.

[22] Kiel C.M., Mikkelsen K.L., Krogsgaard M.R. (2018). Why tibial plateau fractures are overlooked. BMC Musculoskelet. Disord..

[23] Maheshwari J., Pandey V.K., Mhaskar V.A. (2014). Anterior tibial plateau fracture: An often missed injury. Indian J. Orthop..

[24] Alowais S.A., Alghamdi S.S., Alsuhebany N., Alqahtani T., Alshaya A.I., Almohareb S.N., Aldairem A., Alrashed M., Bin Saleh K., Badreldin H.A. (2023). Revolutionizing healthcare: The role of artificial intelligence in clinical practice. BMC Med. Educ..

[25] Pupic N., Ghaffari-Zadeh A., Hu R., Singla R., Darras K., Karwowska A., Forster B.B. (2023). An evidence-based approach to artificial intelligence education for medical students: A systematic review. PLoS Digit. Health.

[26] Xie X., Li Z., Bai L., Zhou R., Li C., Jiang X., Zuo J., Qi Y., Rajasekaran M.P. (2021). Deep Learning-Based MRI in Diagnosis of Fracture of Tibial Plateau Combined with Meniscus Injury. Sci. Program..

[27] Cai D., Zhou Y., He W., Yuan J., Liu C., Li R., Wang Y., Xia J. (2024). Automatic segmentation of knee CT images of tibial plateau fractures based on three-dimensional U-Net: Assisting junior physicians with Schatzker classification. Eur. J. Radiol..

[28] Hobbs D.L., Mickelsen W., Wertz C.I., Stradling C., Boyce M., Chler N., Schneyder D., Jackman C. (2013). Investigating orthogonal radiography in the diagnosis of radial head fractures. Radiol. Technol..

[29] Lind A., Akbarian E., Olsson S., Nåsell H., Sköldenberg O., Razavian A.S., Gordon M. (2021). Artificial intelligence for the classification of fractures around the knee in adults according to the 2018 AO/OTA classification system. PLoS ONE.

